# An Inflammation-Centric View of Neurological Disease: Beyond the Neuron

**DOI:** 10.3389/fncel.2018.00072

**Published:** 2018-03-21

**Authors:** Stephen D. Skaper, Laura Facci, Morena Zusso, Pietro Giusti

**Affiliations:** Department of Pharmaceutical and Pharmacological Sciences, University of Padua, Padua, Italy

**Keywords:** inflammation, mast cells, microglia, astrocytes, oligodendrocytes, neuro-immune, crosstalk, palmitoylethanolamide

## Abstract

Inflammation is a complex biological response fundamental to how the body deals with injury and infection to eliminate the initial cause of cell injury and effect repair. Unlike a normally beneficial acute inflammatory response, chronic inflammation can lead to tissue damage and ultimately its destruction, and often results from an inappropriate immune response. Inflammation in the nervous system (“neuroinflammation”), especially when prolonged, can be particularly injurious. While inflammation *per se* may not cause disease, it contributes importantly to disease pathogenesis across both the peripheral (neuropathic pain, fibromyalgia) and central [e.g., Alzheimer disease, Parkinson disease, multiple sclerosis, motor neuron disease, ischemia and traumatic brain injury, depression, and autism spectrum disorder] nervous systems. The existence of extensive lines of communication between the nervous system and immune system represents a fundamental principle underlying neuroinflammation. Immune cell-derived inflammatory molecules are critical for regulation of host responses to inflammation. Although these mediators can originate from various non-neuronal cells, important sources in the above neuropathologies appear to be microglia and mast cells, together with astrocytes and possibly also oligodendrocytes. Understanding neuroinflammation also requires an appreciation that non-neuronal cell—cell interactions, between both glia and mast cells and glia themselves, are an integral part of the inflammation process. Within this context the mast cell occupies a key niche in orchestrating the inflammatory process, from initiation to prolongation. This review will describe the current state of knowledge concerning the biology of neuroinflammation, emphasizing mast cell-glia and glia-glia interactions, then conclude with a consideration of how a cell's endogenous mechanisms might be leveraged to provide a therapeutic strategy to target neuroinflammation.

## Introduction

Inflammation is a response triggered by damage to living tissues. To quote from *Encyclopædia Britannica, Inc., “*The inflammatory response is a defense mechanism that evolved in higher organisms to protect them from infection and injury. Its purpose is to localize and eliminate the injurious agent and to remove damaged tissue components so that the body can begin to heal.” An inflammatory response can be either acute (seconds to hours) or a response of longer duration referred to as chronic inflammation. The former is usually beneficial, while the latter may result in tissue destruction caused, for example, when regulatory mechanisms of the inflammatory response are defective or when there is an inappropriate immune response with consequent prolonged and damaging inflammation (Castellheim et al., 2009). As succinctly stated by Nathan and Ding (2010), “the core problem with inflammation is not how often it starts, but how often it fails to subside.” Non-resolving inflammation, without doubt, contributes to the overall medical burden in our society, and is now viewed as a new therapeutic frontier (Fullerton and Gilroy, 2016). Inflammation can be especially perilous where the nervous system is involved (so-called “neuroinflammation”), whether it be of an acute nature or chronic—the latter involving sustained activation of glia and recruitment of immune elements. This phenomenon is recognized as a cardinal element in the pathogenesis of both peripheral nervous system conditions like neuropathic pain and other disorders with chronic pain (Myers et al., 2006; Kim et al., 2007; Ellis and Bennett, 2014; Martini and Willison, 2016), and acute (Iadecola and Anrather, 2011) as well as chronic (McGeer and McGeer, 2013; Freeman and Ting, 2016; Ransohoff, 2016a) central nervous system (CNS) diseases, including mood disorders (Najjar et al., 2013; Castanon et al., 2015; Theoharides et al., 2015a; Calcia et al., 2016; Calsolaro and Edison, 2016; Wohleb et al., 2016) and autism (Noriega and Savelkoul, 2014; Theoharides et al., 2016).

Once thought to be immune-privileged, the CNS now enjoys extensive communication links with the immune system. Indeed, were it not for such interactions it is unlikely that neuroinflammation would occur. Immune cell-derived pro-inflammatory mediators play a key role by regulating host responses to infection, inflammation, and reactions to stress or trauma (Le Thuc et al., 2015; Piirainen et al., 2017). While these inflammatory molecules may originate from a number of non-neuronal cell populations, a large body of evidence points to microglia (the brain's main immune guardians) and mast cells, along with astrocytes (and possibly even oligodendrocytes) as important sources of such agents in the above pathologies (Thacker et al., 2007; Appel et al., 2011; Cunningham, 2013; Silver and Curley, 2013; Amor and Woodroofe, 2014; Harcha et al., 2015; Dong et al., 2017; Kempuraj et al., 2017; Skaper et al., 2017; Spangenberg and Green, 2017; Balducci and Forloni, 2018; Simon et al., 2018). Given the complex nature of cellular involvement in inflammation-associated pathologies across the central and peripheral nervous systems, viewing neuroinflammation in the context of microglia (Masgrau et al., 2017), astrocyte or mast cell involvement alone fails to fully appreciate the homotypic and heterotypic cell—cell interactions that are an integral part of the inflammation process. This review is intended to describe recent contributions in our understanding of the biology and cellular signaling mechanisms of inflammation as it affects the nervous system, with emphasis on a mast cell-glia (microglia, astrocyte, oligodendrocyte) interactions. This will be followed by a consideration of approaches to counteract neuroinflammation that capitalize on natural defense mechanisms and lipid signaling molecules.

## Glia

Among the cell types that participate in inflammation of the nervous system, tissue-resident and blood-borne glia and immune system-derived cells comprise key elements. Microglia are the principal immune effector cells of the brain, constantly surveying their environment in preparation for insult or injury (“immunosurveillance”). When activated, they phagocytose cellular debris, present antigens to T cells and release cytokines/chemokines, the latter providing cells with the ability to communicate with one another and orchestrate complex multicellular behavior (Becher et al., 2017). In homeostatic terms, microglia regulate cell death and neurogenesis, and actively engulf synaptic material and play a major role in synaptic pruning during postnatal development, thereby linking microglia surveillance to synaptic maturation (Paolicelli et al., 2011). This synaptic pruning is dependent upon neural activity and the microglia-specific phagocytic signaling pathway, complement receptor 3/C3 (Schafer et al., 2012). Interestingly, this normal developmental synaptic pruning pathway appears to be activated early in the Alzheimer disease (AD) brain and mediates synapse loss (Hong et al., 2016). Yet another role for microglia in CNS development comes from a new study describing a unique phenotype of neonatal (CD11c^+^) microglia in primary myelinating areas of the developing brain that deliver signals necessary for myelination and neurogenesis (Wlodarczyk et al., 2017).

**Figure 1:**
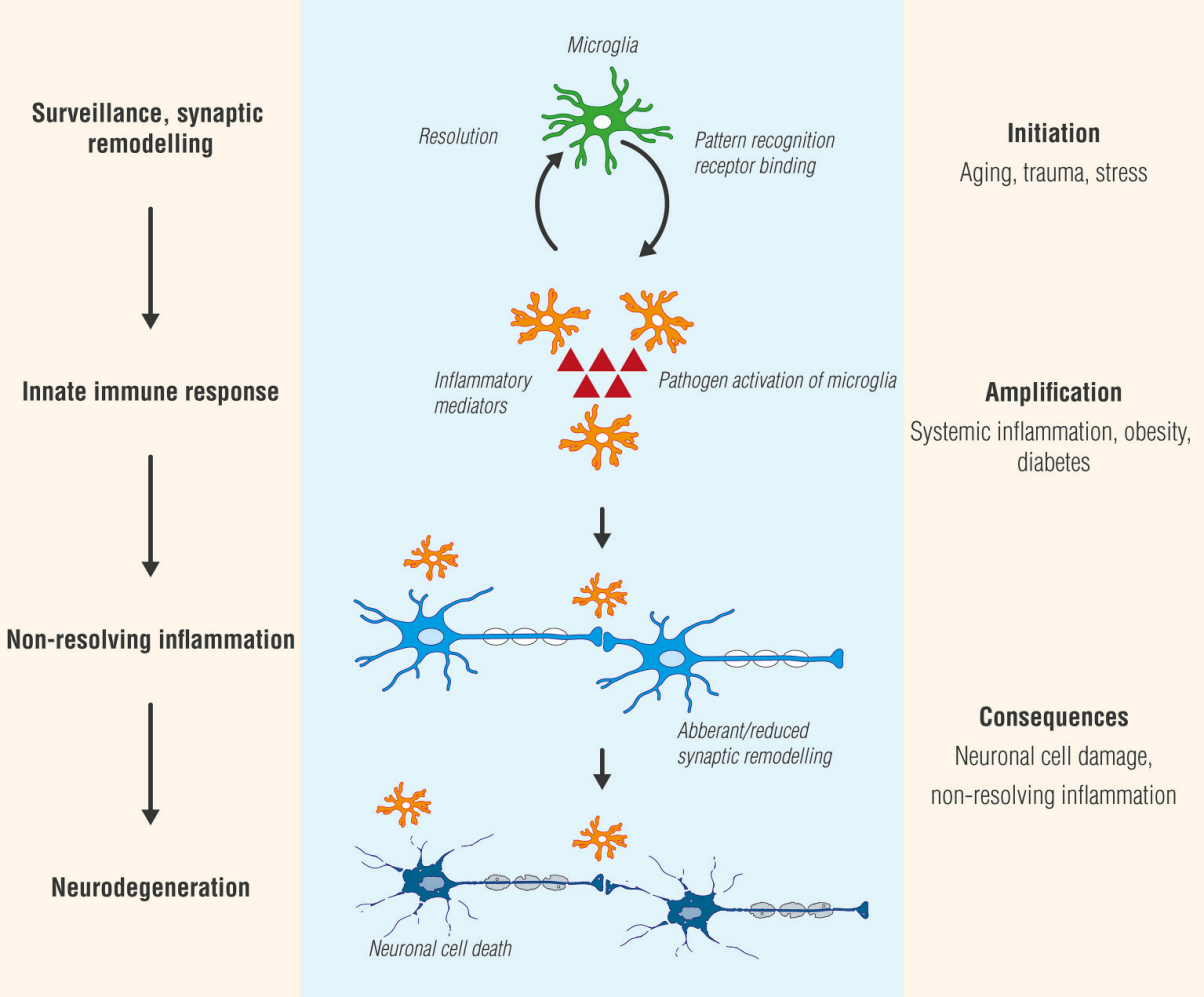
Microglia, like Janus, the two-faced Roman god of beginnings and transitions, display two sides—physiological as well as pathological. While microglial cell activation participates in surveillance that functions to maintain homeostasis and promote synaptic maturation, prolonged exposure to pathogen activators or in settings of systemic inflammation, as may occur in conditions such as diabetes or obesity, can culminate in a state of chronic, non-resolving neuroinflammation. Ultimately, these responses will provoke functional and structural changes and neuronal cell death (neurodegeneration).

Microglia plasticity is complex, and activation states have generally been classified into two functional subtypes: M1 (classic/pro-inflammatory) and M2 (alternative polarization/neuroprotective) (Tang and Le, 2016). While providing a framework for exploring the diverse functions of microglia, this terminology has been questioned (Ransohoff, 2016b). At least in the case of experimental autoimmune encephalomyelitis (EAE), a widely utilized animal model of MS, inhibiting inflammatory agent secretion by microglia reduces disease severity, while transplanting M2 polarized microglia into the CNS facilitated recovery (Miron et al., 2013; Zhang et al., 2014). Studies by Peferoen et al. (2014) indicate that microglia express an intermediate activation status in all pre- active and remyelinating MS lesions and distinct from microglia profiles in actively demyelinating lesions, thus supporting the view that activation status of microglia is a dynamic process which occurs as a continuum across the M1 and M2 phenotypes. Microglial cell progression from beneficial to detrimental is schematically portrayed in Figure 1.

**Figure 2:**
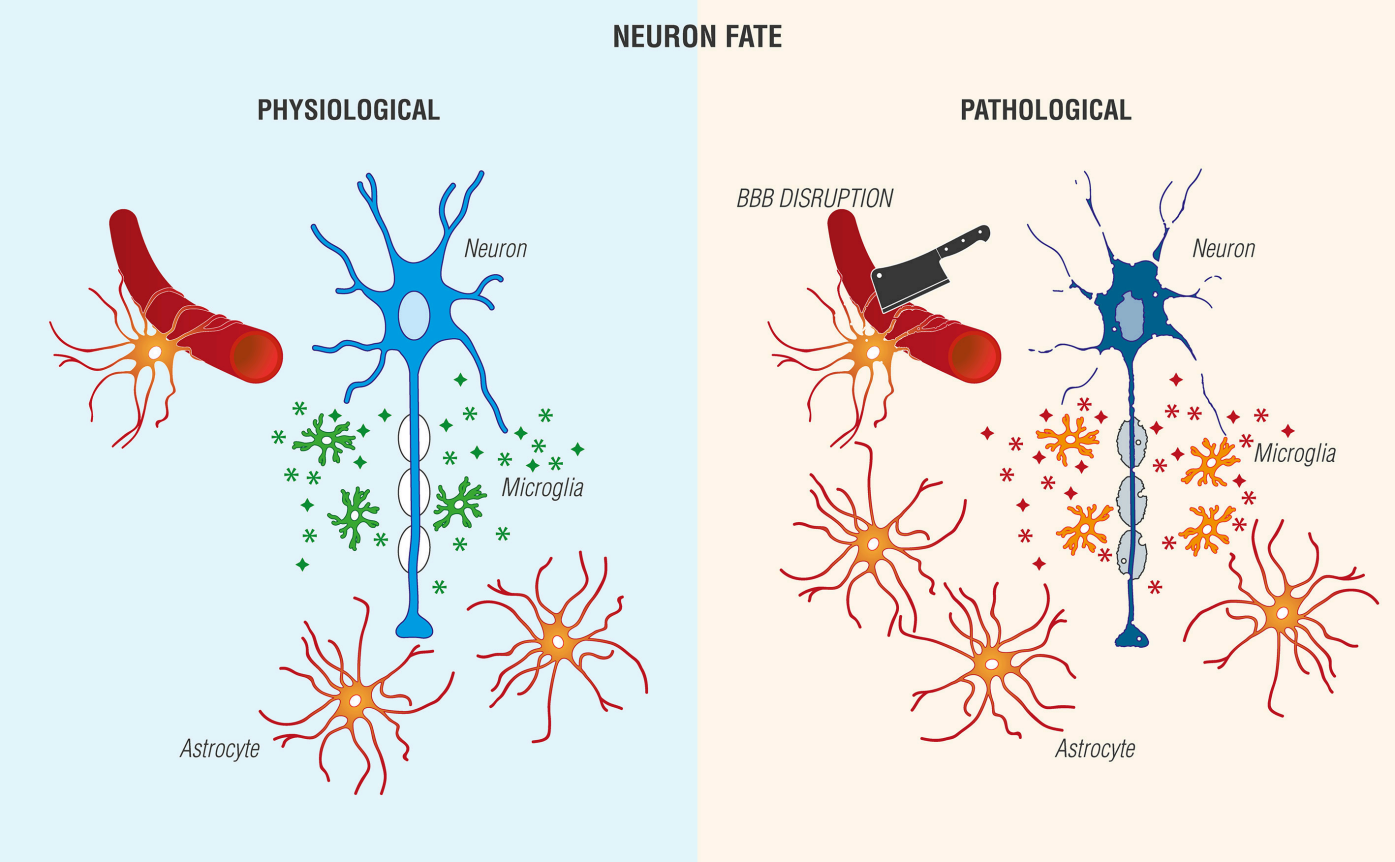
Reciprocal interactions between microglia and astrocytes provoke beneficial and harmful effects in the brain. **(Left)** Physiological actions include microglia phagocytosis/debris clearance, release of anti-inflammatory cytokines/chemokines (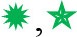), and trophic agents to favor neuronal cell survival. **(Right)** Non-resolving neuroinflammation results in a pathological, pro-inflammatory activation profile of microglia/mediator production (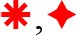), blood-brain barrier (BBB) compromise, immune cell infiltration, gliosis, and neuronal cell death [Adapted and extensively modified from Le Thuc et al. (2015). The complex contribution of chemokines to neuroinflammation: switching from beneficial to detrimental effects (Figure 3). Copyright © 2015 John Wiley and Sons. With permission].

Astrocytes, the most abundant cell type in the CNS and historically viewed as “brain glue,” participate in a number of critical physiological functions, including maintaining blood-brain barrier (BBB) integrity by forming astrocytic end feet around endothelial cells, regulation of axonal outgrowth and myelination (Kiray et al., 2016), and formation of intracellular communication networks, e.g., signaling via Ca^2+^ release and uptake (Volterra and Meldolesi, 2005; Kimelberg and Nedergaard, 2010). Injury leads to an increase in astrocyte reactivity (Sofroniew and Vinters, 2010) with changes in morphology, increased expression of glial fibrillary acidic protein, proliferation and secretion of pro-inflammatory molecules and growth factors (Jensen et al., 2013; Pekny et al., 2016). Further, these factors may exert autocrine/paracrine actions to promote astrocytic reactivity and impact neighboring cells. Astrocyte-microglia interactions are also possible (Figure 2).

Oligodendrocytes (OLs) are responsible for myelin production in the CNS, and are generated in the germinal zone from migratory bipolar oligodendrocyte precursor cells (OPCs; Grinspan, 2002). Myelinating OLs not only provide trophic support for axons, but also release lactate through the monocarboxylate transporter 1 which is then utilized by axons for mitochondrial ATP generation (Saab et al., 2013). The migration of OPCs is influenced by receptor-ligand adhesions with the extracellular matrix, such as integrins, and signaling molecules (Soliven, 2001) which may provide a critical link between neuronal cell activity and OPCs. Cells of the OL lineage acquire cell surface markers with maturation and respond specifically to factors which regulate proliferation, migration, differentiation, and survival. Moreover, cells of the OL lineage express and respond to a broad range of receptor-ligand pairs, including glutamic acid, γ-aminobutyric acid, ATP, serotonin, acetylcholine, nitric oxide, opioids, prostaglandins, prolactin, cannabinoids, and nuclear receptors (Marinelli et al., 2016). While known for their ability to support axonal functions and long-term integrity (Nave, 2010), OLs participate also in neuropathology, as will be discussed in later sections.

## Mast cells

Glial cell participation in inflammation-associated neuropathologies encompasses not only their inflammatory signals, but also their response to mediators produced by other immune system-derived cells, both blood-borne (dendritic cells, lymphocytes, neutrophils), and tissue-resident (mast cells). While receiving comparably less attention than glia, the mast cell nonetheless represents an important peripheral immune signaling link to the brain in an inflammatory setting. Mast cells share a relationship with basophils but have a distinct haematopoietic lineage development, leaving the circulation to enter peripheral tissues where the local environment determines protease phenotype expression packaged in cytoplasmic granules; these also contain histamine and heparin (Table 1; Prussin and Metcalfe, 2003). As a major sensory arm of the body's innate immune system, mast cells function as environmental “sensors” to communicate with other elements in physiological and/or immune responses thanks to their widespread tissue presence near blood vessels and surfaces exposed to the environment (Gilfillan et al., 2011). These immune effector cells are found in tissues innervated by small caliber sensory nerve fibers (A-delta and C-fibers responsible for pain transmission that extend from the periphery to the spinal cord and brain), in meninges, and apposing cerebral blood vessels. Upon activation, mast cells secrete “packaged” or synthesized *de novo*, numerous vasoactive, neurosensitizing and pro-inflammatory mediators, which include biogenic amines (histamine, serotonin), cytokines, proteolytic enzymes (e.g., chymase, tryptase, acid hydrolases, among others), lipid metabolites (prostaglandin D2, leukotriene C4, platelet-activating factor), ATP, neuropeptides, nerve growth factor (NGF), vascular endothelial growth factor and nitric oxide (Kalesnikoff and Galli, 2008; Silver and Curley, 2013). While more than 50 mediators are known to date, their expression by mast cells is heterogeneous and determined to a large extent by species and tissue location. Additionally, mast cell-derived chemoattractants recruit eosinophils (Wardlaw et al., 1986), monocytes, and neutrophils (Wezel et al., 2015).

**Table 1:** Mast cells: a primer.

**Origin and classification:**
• First description in 1878, noted for their staining characteristics and abundant cytoplasmic granules • Share some characteristics with circulating basophil granulocytes; thought to arise from distinct bone marrow precursor cells expressing CD34 • Unique hematopoietic lineage development in comparison to other myeloid-derived cells: immature lineage mast cells leave the bone marrow to enter the circulation and immediately undergo transendothelial recruitment into peripheral tissues where formation of secretory granules with a particular protease phenotype is regulated by the peripheral tissue. • Mast cell types are generally divided into connective tissue cells and a distinct set, mucosal mast cells (whose activities are dependent on T-cells) • Broad tissue distribution, often close to blood vessels and prominent near boundaries between the body's external environment and the internal milieu, such as skin, mucosa of lungs and digestive tract, and in mouth, conjunctiva, and nose • Mast cells also found in the nervous system, including meninges, brain parenchyma, and nerve endoneurium
**Physiology:**
• Play a key role in the inflammatory process • Upon activation rapidly release mediator-loaded granules into the interstitium • Degranulation is caused by direct injury (e.g., physical or chemical), cross-linking of IgE receptors or by activated complement proteins • Elaborate a vast array of important cytokines and other inflammatory mediators • Express multiple “pattern recognition receptors” (e.g., Toll-like receptors) involved in recognizing broad classes of pathogens • Granules loaded with a plethora of bioactive chemicals, proteoglycans, serine proteases, neuropeptides, and growth factors; can be transferred to nearby immune cells and neurons via transgranulation and their pseudopodia
**Disease involvement:**
• Allergic reactions • Anaphylactic shock • Inflammatory pain, chronic (including neuropathic) pain • Acute and chronic neurodegenerative disorders • Mood disorders

As antigen-presenting cells, mast cells can induce T cell activation, proliferation, and cytokine secretion (Bulfone-Paus and Bahri, 2015). Indeed, the capability of mast cells to present antigens by class I and II major histocompatibility complex molecules, respective, to CD4^+^ and CD8^+^ T cells constitutes a major antigen-dependent interaction between mast cells and T cells—the so-called immunological synapse (Monks et al., 1998; Grakoui et al., 1999; Suurmond et al., 2013), and depends on cytoskeletal control of receptor triggering (Comrie and Burkhardt, 2016). Optimal activation of antigen-specific T cells requires interaction between CD28 on T cells and CD86/CD80 on mast cells. Additional interaction between mast cell OX40L and T cell OX40—together with mast cell-derived tumor necrosis factor-α (TNF-α)–promotes antigen-stimulated mast cell enhancement of T cell activation (Nakae et al., 2006) while polarizing T cell secretory machinery toward the mast cell (Gaudenzio et al., 2009). It is not surprising, thus, to see mast cell involvement in T cell-associated immune responses such as EAE (Elieh Ali Komi and Grauwet, 2017).

## Neuroinflammation is amplified by mast cell—glia and glia—glia crosstalk

The contribution of mast cells and glia to neuroinflammation is strongly influenced by their potential for mutual interaction and exacerbation of pathology. These cell types are often found in close proximity to each other, facilitating cell-cell communication. Further, ligand-receptor pairings, whose expression may be up-regulated in inflammatory tissues, can facilitate chemotactic actions to bring mast cells and glia in closer contact. Indeed, recruitment and activation of these immune cell populations in a defined temporal pattern necessitates a reciprocal communication between them. Some examples are briefly discussed below.

The complement system appears to play a role in crosstalk between mast cells, microglia, and astrocytes. For example, microglia (and astrocytes) showed up-regulation of the chemoattractant anaphylatoxin peptide C5a and its receptor CD88 in inflamed CNS tissues (Gasque et al., 1997). Complementary expression of C5a receptor on activated mast cells provides a strong chemoattractant signal toward C5a peptide (Pundir et al., 2015). The C5a-C5a receptor pathway plays a vital role in brain inflammatory injury, including intracerebral hemorrhage (Young et al., 2013; Yuan et al., 2017). In the context of AD, the central complement factor C3 secreted from astrocytes interacts with microglial C3a receptor to mediate β-amyloid pathology and neuroinflammation in AD mouse models (Lian et al., 2015, 2016). Neuronal cell overproduction of Aβ activates astroglial nuclear factor-κB (NF-κB) to elicit extracellular release of C3.

**Figure 3:**
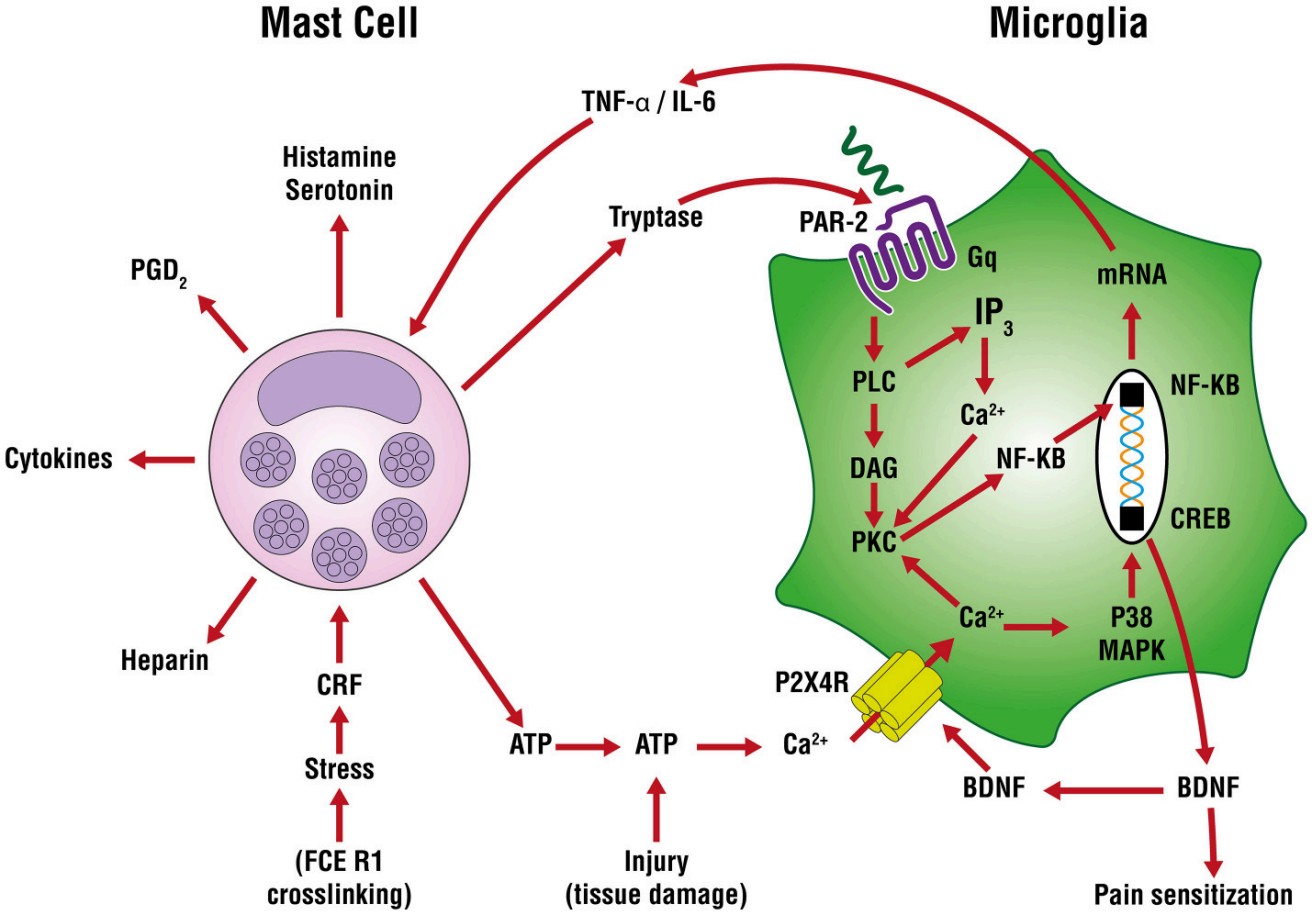
Mast cell—microglia crosstalk in the release of brain-derived neurotrophic factor (BDNF). ATP-induced BDNF expression and release is mediated by the P2X_4_ receptor through a mechanism involving Ca^2+^ entry, induction of Ca^2+^/inositol 1,4,5-trisphosphate/PKC signaling, phosphorylation of IKKα and IKKβ and activation and nuclear translocation of nuclear factor-κB (NF-κB) and gene induction The purinergic P2X_4_ receptor acts to release BDNF via mast cell tryptase cleavage/activation of protease-activated receptor 2 (PAR2) on microglia which couples to G proteins and induces canonical phospholipase C (PLC)/Ca^2+^/protein kinase C (PKC) signaling, activation and nuclear translocation of NF-κB, culminating in BDNF gene induction and translation. The latter cells release tumor necrosis factor-α (TNF-α) and interleukin-6 (IL-6) which can further drive mast cell activation and degranulation and numbers, leading to a potential feedback loop between mast cells and microglia.

In neuropathic (and other forms of chronic pain) pain, plastic changes in dorsal horn neurons contribute to a phenomenon of hypersensitivity to pain sensation that is maintained over time, known as central sensitization (Constandil et al., 2011). The neurotrophin brain-derived neurotrophic factor (BDNF) is a crucial neuromodulator involved in nociceptive hypersensitivity in the CNS (Coull et al., 2005; Khan and Smith, 2015). BDNF generates a long-lasting neural excitability change in the spinal cord via tyrosine kinase B receptor signaling, similar to that observed in chronic pain models such as neuropathy (Constandil et al., 2011). Peripheral nerve injury up-regulates the purinergic P2X_4_ receptor in microglia of the sensory part of the spinal cord (Beggs et al., 2012) to mediate BDNF release and neuropathic pain (Ulmann et al., 2008). An important signaling pathway in the development of neuropathic pain is extracellularly-derived ATP, a ubiquitous danger signal released from damaged cells which engages P2 purinoceptors on target cells (Burnstock, 2016). ATP is a potent stimulus for microglia, as well. Distinct P2 receptor subtypes are expressed on mast cells as a function of species and source from which mast calls are derived (Bulanova and Bulfone-Paus, 2010). ATP has a rather large “footprint” and, once released from a mast cell (e.g., FcεR1 cross-linking, stress) can diffuse several hundred micrometers to act on in neighboring cells, including other mast cells as an autocrine/paracrine factor (Osipchuk and Cahalan, 1992). ATP-induced BDNF expression and release is mediated by the P2X_4_ receptor (Klein et al., 2012) through a mechanism involving Ca^2+^ entry, induction of Ca^2+^/inositol 1,4,5-trisphosphate/PKC signaling, phosphorylation of IKKα and IKKβ and activation and nuclear translocation of NF-κB and gene induction (Trang et al., 2009; Figure 3). Another route by which P2X_4_ receptor acts to release BDNF involves mast cell tryptase cleavage of microglial protease-activated receptor 2 (PAR2) which couples to G proteins and induces canonical phospholipase C/Ca^2+^/protein kinase C signaling that leads to the activation and nuclear translocation of NF-κB and gene induction (Yuan et al., 2010; Sakamoto et al., 2016; Figure 3). Microglia, by releasing pro-inflammatory cytokines like TNF-α and interleukin-6 (IL-6), amplify mast cell activation/degranulation and numbers (Zhang et al., 2011). An additional mast cell-microglia feedback loop, again linked to ATP, invokes its binding to P2 receptors to stimulate release of IL-33 from microglia pre-activated with pathogen-associated molecular patterns (PAMPs) acting on Toll-like receptors (TLRs), thereby inducing mast cell secretion of IL-6, IL-13, and CCL2 which then modulate microglia activity.

Cytokines released following binding of PAMPs to TLR2/TLR4 on mast cells recruits immune cells (e.g., microglia) to the sites of injury that is dependent on signaling pathways involving TLR2/TLR4 (Pietrzak et al., 2011). Further, mast cell activation up-regulates chemokine expression (including CCL5/RANTES); the latter molecules are capable of inducing a pro-inflammatory response in microglia. Microglia-derived IL-6 and CCL5 may, in turn, influence mast cell expression of TLR2/TLR4.

Activation of antigen-presenting cells, including mast cells, requires the co-stimulatory protein CD40 for binding to CD40L. A CD40-CD40L interaction has been described between mast cells and astrocytes which induces cytokine and chemokine production via Rho-family GTPases/Ca^2+^-dependent protein kinase C isoforms, mitogen-activated protein kinases, NF-κB, and signal transducer and activator of transcription 1 (Kim et al., 2011). These authors observed also a feedback action of the released cytokines on astrocytes to bring about their re-activation. Additional microglia—astrocyte interactions have been demonstrated at the level of translocator protein, a marker of gliosis in neurodegeneration (Wang et al., 2014) and in terms of their ability to exercise a reciprocal interaction through release of pro-inflammatory cytokines/chemokines (Le Thuc et al., 2015). Moreover, several investigators have proposed a crosstalk between oligodendrocytes and microglia involving exosomes—small vesicles containing proteins, lipids, and regulatory RNAs thought to provide a means of intercellular communication and of transmission of macromolecules between cells via membrane vesicle trafficking, thereby influencing the immune system (Peferoen et al., 2014) (Table 2).

**Table 2:** Avenues of mast cell—glia and glia—glia communication.

	**Biological actions**		
**Effector**	**Microglia/Astrocytes/Oligodendrocytes**	**Mast cells**	**References**
C5a receptor (C5aR)	C5aR up-regulated by microglia activation; C5a peptide released in neuroinflammation; crosstalk between C5a and TLR4	C5aR up-regulated by activation; strong mast cell chemoattractant signal toward C5a peptide; crosstalk between C5a and TLR4	Gasque et al., 1997; Griffin et al., 2007; Soruri et al., 2008; Yuan et al., 2017
C3 and C3 receptor (C3aR)	Astrocyte C3 interacts with microglial C3aR to mediate Aβ pathology and neuroinflammation		Lian et al., 2015, 2016
ATP/P2 receptors	ATP stimulates IL-33 release from microglia pre-activated with pathogen-associate molecular patterns via TLRs	IL-33 binds to mast cell receptor to induce secretion of IL-6, IL-13 and monocyte chemoattractant protein 1 which modulate microglial cell activity	Osipchuk and Cahalan, 1992; Burnstock, 2016
Proteinase-activated receptor 2 (PAR2)	Mast cell tryptase cleaves/activates PAR2 on microglia, resulting in P2X_4_ receptor up-regulation and release of brain-derived neurotrophic factor	Microglial cell IL-6 and TNF-α up-regulate mast cell expression of PAR2, with mast cell activation and TNF-α release	Osipchuk and Cahalan, 1992; Yuan et al., 2010; Zhang et al., 2010
TLR2, TLR4	Microglial cell-derived IL-6 and CCL5 affect mast cell expression of TLR2/TLR4	Up-regulation of cytokine/chemokine release; CCL5/RANTES induces pro-inflammatory profile in microglia; recruitment of immune cells (including mast cells) to site(s) of injury	Orinska et al., 2005; Tanga et al., 2005; Kim et al., 2007; Buchanan et al., 2010; Pietrzak et al., 2011; Skuljec et al., 2011; Liu et al., 2012
CXCR4/CXCL12	Promotion of microglia migration/activation; CXCR4/CXCL12 up-regulated in hypoxia/ischemia	CXCR4 is a mast cell chemotaxin	Juremalm et al., 2000; Yang et al., 2010; Knerlich-Lukoschus et al., 2011
CD40/CD40L	CD40 up-regulated on activated astrocytes; crosstalk with CD40L leads to production of inflammatory cytokines/chemokines trigger mast cell degranulation	CD40L expression enhanced in activated mast cells; crosstalk with CD40 leads to production of inflammatory cytokines	Kim et al., 2011
IL-33	Released by oligodendrocytes in neuropathic pain	IL-33 binds to mast cell receptor to induce secretion of TNF-α which up-regulates oligodendrocyte expression of acute phase proteins	Zarpelon et al., 2016; Barbierato et al., 2017
Serum amyloid A (SAA)	Up-regulated by inflammatory cytokines released from glia, mast cells; localized to amyloid β-peptide deposits	SAA is an attractant for mast cells; mast cell-derived cytokines (e.g., TNF-α can uo-regulate SAA expression by glia	Nelson et al., 1993; Olsson et al., 1999; Barbierato et al., 2017
Translocator protein (TSPO)	Retinal inflammation/injury leads to TSPO up-regulation in retinal microglia; TSPO endogenous ligand diazepam-binding inhibitor (DBI) up-regulated in microglia; DBI-TSPO signaling promotes microglia-microglia interactions		Wang et al., 2014
Cytokines/chemokines (e.g., CCL2)	Reciprocal interactions between microglia and astrocytes		Le Thuc et al., 2015; Xu et al., 2017
Exosomes	Participate in oligodendrocyte—microglia crosstalk		Peferoen et al., 2014
IL-18/IL-18 receptor	Nerve injury increases expression of IL-18 in microglia and IL-18R in astrocytes; IL-18 induces astrocytic hypertrophy and release of IL-1β, IL-6, TNF-α		Miyoshi et al., 2008

*IL, interleukin; TLR, Toll-like receptor; TNF-α, tumor necrosis factor-α*.

## Inflammation and neurological disorders

### Alzheimer disease

Alzheimer disease is the most prevalent chronic, progressive neurodegenerative disease, and cause of dementia (Selkoe and Hardy, 2016). Principal pathological features are the presence in brain of focal extracellular deposits (senile plaques) of fibrillar amyloid β-peptide (Aβ) and intracellular neurofibrillary tangles composed of hyperphosphorylated tau protein (Selkoe, 2011). The amyloid hypothesis of AD was initially based on the idea that pathogenesis starts with amyloid deposition as senile plaques, although this view has shifted to AD as a disease in which soluble oligomeric forms of Aβ impair synaptic plasticity and behavior (Selkoe, 2008). Disease pathogenesis involves also interactions with immunological mechanisms in the brain. Regional inflammatory responses occur in AD brain with deposits of Aβ as foci, with elevated expression of pro-inflammatory cytokines, acute phase proteins, and complement components (Cameron and Landreth, 2010; Calsolaro and Edison, 2016), along with signs of activated microglia and reactive astrocytes (Wyss-Coray, 2006; Medeiros and LaFerla, 2013; Regen et al., 2017; Balducci and Forloni, 2018). Post-mortem AD brain samples and those of mouse transgenic models of AD bearing deposits of insoluble Aβ display alterations in microglia and astrocytes (Parvathenani et al., 2003; Heneka et al., 2015); Aβ plaques in the frontal cortex of AD brain are surrounded by IL-1β-positive microglia (Heuberger, 2011). Amyloid precursor protein, from which Aβ peptides derive reportedly regulates microglial phenotype (Manocha et al., 2016). In AD brain, activated microglia may phagocytose toxic Aβ and produce survival-promoting trophic factors (Rivest, 2009); however, if prolonged such activation can result in the elaboration of synaptotoxic/neurotoxic cytokines, chemokines, and reactive oxygen/nitrogen species (Wyss-Coray, 2006; Rivest, 2009; Balducci and Forloni, 2018; Simon et al., 2018). Indeed, Biscaro et al. (2012) using a transgenic mouse AD model demonstrated that inhibition of microglial activation protects hippocampal neurogenesis and improves cognitive deficits.

Recent studies have defined a complement-dependent intercellular cross talk whereby Aβ overproduction by neurons activates astroglial cell NF-κB to elicit release of C3 (Lian et al., 2015, 2016). The released C3, in turn, interacts with neuronal and microglial cell C3a receptor (C3aR) to alter cognitive function and impair Aβ phagocytosis—in effect, promoting a pathogenic cycle. Abnormal activation of NF-κB has been implicated in AD (Kaltschmidt et al., 1997), and Lian et al. (2015) showed that exposure to Aβ activates astroglial NF-κB and C3 release.

A role for mast cells in AD disease onset/progression is less well-established. Tryptase-positive mast cells were found in proximity to Aβ plaques in post-mortem AD brain (Maslinska et al., 2007), and fibrillar Aβ was reported to cause CD47-dependent mast cell secretory and phagocytic responses (Niederhoffer et al., 2009). In a more recent study, Harcha et al. (2015) showed that acute treatment of brain mast cells with the 25–35 amino acid fragment of Aβ activates Panx1 and Cx43 hemichannels, accompanied by increases in Ca^2+^ influx, degranulation, and histamine release. These authors also found raised Panx1 and Cx43 hemichannel activity in a transgenic mouse AD model along with greater mast cell numbers in cortex and hippocampus prior to Aβ plaque formation. Acute phase proteins like serum amyloid A (SAA) are produced in response to inflammation. SAA immunoreactivity co-localized with Aβ deposits in AD brain (Kindy et al., 1999), and SAA concentration was much higher in cerebrospinal fluid (CSF) of AD subjects than in normal controls (Miida et al., 2006). A new study by Barbierato et al. (2017) shows that inflammatory stimuli up-regulate expression of SAA1 by CNS glia—including oligodendrocytes—providing an attractant for mast cells and promoting movement toward Aβ deposits (Nelson et al., 1993; Olsson et al., 1999). The data propose a potential for paracrine/autocrine effects between these cell types. Moreover, Harcha et al. (2015) suggest that mast cells are among the first brain cells to sense Aβ peptides and thus may play a critical role in the onset and progression of AD. Masitinib, a potent and selective oral protein tyrosine kinase inhibitor targeting c-Kit (the receptor for the mast cell growth factor stem cell factor; Dubreuil et al., 2009) that inhibits the survival, migration and activity of mast cells, when administered as add-on therapy to AD patients receiving standard care during 24 weeks was associated with slower cognitive decline (Piette et al., 2011). It is currently in Phase II/III clinical trials for the treatment AD (Folch et al., 2015).

Neuroendocrine and behavioral changes accompanying the stress response can affect homeostasis, over the long term, in terms of detrimental effects such as impairing neuronal cell metabolism, plasticity, and survival. Stress-induced hormonal and behavioral reactions may also indirectly induce neuropathological processes participating in the development and progression of AD (Mravec et al., 2018), in part through mast cell-mediated BBB breakdown (Esposito et al., 2001). Indeed, chronic stress reportedly accelerates AD pathogenesis in human and animal models through increases in inflammatory responses, Aβ accumulation, tau hyperphosphorylation, oxidative stress, mitochondrial impairment, and glucose metabolism (Machado et al., 2014), while early-life stress increases the risk of cognitive disorders in an aged mouse model of AD (Hoeijmakers et al., 2017). Mast cell activation plays a crucial role in stress-dependent inflammatory mechanisms. Human mast cells synthesize and secrete corticotropin releasing hormone (CRH) and express functional CRH receptors (Cao et al., 2005). CRH released from mast cells can act in an autocrine/paracrine manner to activate mast cells and microglia in stress and neuroinflammatory conditions (Karagkouni et al., 2013; Kritas et al., 2014a). Chronic psychological stress is a risk factor for dementia and AD by inducing microglial proinflammatory status (Piirainen et al., 2017) and, conceivably, a crosstalk loop between the former cells and mast cells. These observations propose that mast cells play a crucial role in stress responses associated with inflammation that may predispose to AD pathogenesis in high-risk groups.

### Parkinson disease

Parkinson disease (PD) is the first and second most prevalent motor and neurodegenerative disease, respectively (Hirsch et al., 2016). Pathological hallmarks of PD are the progressive death of dopaminergic neurons in the substantia nigra pars compacta and intracellular accumulation of Lewy bodies enriched in α-synuclein protein (Shulman et al., 2011). In addition to motor defects, clinical features of PD comprise non-motor symptoms that become increasingly prevalent during the course of the disease. Although PD is a complex, multisystem disorder, neuroinflammatory responses, and neuroinflammation appear to exacerbate PD pathogenesis (Stojkovska et al., 2015; Wang et al., 2015). A number of studies suggest that microglial cell activation may have a role in PD. For example, Zhang et al. (2017) reported that pathological α-synuclein exacerbates progression of PD through microglial activation via the transcription factor NF-κB and expression of pro-inflammatory cytokines such as TNF-α and IL-1β. Further, expression of major histocompatibility complex II by microglia is needed for activation of these cells by α-synuclein, which can play a role in immune responses (Harms et al., 2013). A number of gene defects have been identified in familial forms of PD, the most commonly mutated gene being that for leucine-rich repeat kinase 2, whose pathogenic mutations influence the ability of microglia to internalize and degrade α-synuclein—thereby exacerbating α-synuclein-induced microglial pathology and neuroinflammation (Recchia et al., 2004; Schapansky et al., 2015). Immunomodulator dysregulation increases microglial activation and the degeneration of dopamine neurons (Zhang et al., 2011). Dopaminergic neurons are especially sensitive to injury by pro-oxidant species, and whose activation of microglia in PD can lead to degeneration of dopaminergic cells (Appel et al., 2010; Herrera et al., 2015).

A role that mast cells may play in PD pathogenesis is, until now, largely lacking. However, recent reports by Kempuraj et al. (2016, 2018) showed that incubation of mouse bone marrow-derived mast cells (BMMCs) and human umbilical cord blood-derived cultured mast cells with the dopaminergic toxin 1-methyl-4-phenylpyridinium (MPP^+^) led to release of the chemokine CCL2 and matrix metalloproteinase-3 which is claimed to play a role in PD pathogenesis. Moreover, MPP^+^-treated BMMCs exposed to glia maturation factor (an activator of glia inducing neuroinflammation/neurodegeneration; Zaheer et al., 2007) enhanced CCL2 release. Interestingly, MPP^+^-induced CCL2 release was greater in BMMCs-astrocyte co-cultures (see also Kim et al., 2010). Based on their findings the authors suggest that mast cells may play role in PD pathogenesis.

### Multiple sclerosis

Multiple sclerosis (MS) is the prototypical inflammatory disease of the CNS, whose defining feature is the destruction of myelin (Compston and Coles, 2008). MS is the most frequent cause of chronic neurological impairment in young people (Kamm et al., 2014). Often thought of as a disorder of white matter demyelinating lesions, cortical demyelination contributes greatly to MS disabilities and may even precede the appearance of classic white matter plaques in some MS patients (Lucchinetti et al., 2011). The autoimmune nature of the disease (whether through genetic predisposition or yet to be elucidated environmental triggers) is initiated by myelin-reactive T cells, being then amplified by an inflammatory response involving myeloid cells, including microglia and infiltrating macrophages (Peferoen et al., 2015; Sospedra and Martin, 2016). Triggering receptor expressed on myeloid cells 2 (TREM-2) is a member of the immunoglobulin and lectin-like superfamily and operates as part of the innate immune system. It is highly expressed by microglia (Hickman et al., 2013) and facilitates nervous tissue debris clearance (Takahashi et al., 2007). TREM-2 is cleaved by microglia to produce soluble TREM-2, whose levels are reportedly increased in CSF of patients with relapsing-remitting, secondary progressive, and primary progressive MS, being normalized by treatment with the immunomodulatory drug natalizumab (Öhrfelt et al., 2016).

Mast cells are likely to play an important role in MS pathogenesis (Theoharides et al., 2008b; Kritas et al., 2014b; Conti and Kempuraj, 2016) by mediating inflammation and demyelinization via presentation of myelin antigens to T cells and/or disrupting the BBB, thereby allowing entry of inflammatory cells and cytokines. In the latter instance, expression of the mast cell chemoattractant CXCL12 at the lumen surface of endothelial cells (McCandless et al., 2008) could favor trafficking and accumulation of CXCR4-expressing mast cells. Mast cell tryptase is elevated in CSF from MS patients (Rozniecki et al., 1995), promotes mononuclear cell secretion of TNF-α and IL-6 (Malamud et al., 2003), and stimulates protease-activated receptors (PARs) that may disrupt BBB integrity (Bunnett, 2006). Interestingly, barrier breakdown occurs prior to pathological/clinical signs of MS (Kermode et al., 1990). EAE, a widely utilized animal model of MS based on an immune reaction against myelin oligodendrocyte glycoprotein, evidences degranulating mast cells in brain (Brenner et al., 1994). Mast cell activation and neutrophil recruitment is reported to promote early and robust inflammation in the meninges in EAE (Christy et al., 2013). Myelin activates mast cells (Medic et al., 2008), causing demyelination (Theoharides et al., 1991) and oligodendrocyte cell death (Medic et al., 2010). In spite of the broad use of these mouse EAE models, disagreement remains as to whether mast cell effects on EAE development depend on mouse strain, immunization protocol, or disease type and severity (Nelissen et al., 2013).

Mast cell-derived IL-1 and IL-6 promote transition of regulatory T cells to IL-17-producing active T helper type 17 (Th17) lymphocytes (Dudeck et al., 2011; El-Behi et al., 2011; Ganeshan and Bryce, 2012). IL-17 is elaborated by both adaptive immune cells (e.g., Th17 and cytotoxic T cells; Kolbinger et al., 2016) and innate immune cells (e.g., mast cells; Kan et al., 2016). By synergizing with other pro-inflammatory cytokines (e.g., released by microglia and mast cells themselves), IL-17 can induce release of yet additional cytokines/chemokines to recruit new inflammatory cells, ultimately impacting the function of microglia, astrocytes, oligodendrocytes, neurons, neural precursor cells and endothelial cells (Kolbinger et al., 2016).

The Th17 cytokine granulocyte macrophage-colony stimulating factor (GM-CSF) is a key player in EAE-associated neuroinflammation, as demonstrated by the absence of myelin-specific T cell accumulation in meninges and production of GM-CSF in mast cell-deficient animals (Russi et al., 2016a,b). Using mast cell-T cell co-cultures and selective mast cell reconstitution of the meninges of mast cell-deficient mice, these authors showed meningeal mast cells to be an early source of caspase-1-dependent IL-1β production. IL-1β promotes T cell expression of GM-CSF, thereby enhancing their encephalitogenicity. Interestingly, MS patients in the effector phase display mast cell-T cell co-localization (crosstalk?) in the meninges and CNS (Russi et al., 2016b). Beyond white matter demyelination, plaques in the gray matter also contribute to MS disease pathogenesis, with cortical demyelination being characterized by inflammation in the meninges—where mast cells are resident.

Another member of the IL-1 cytokine family, IL-33, is tied in to inflammatory and autoimmune diseases (Liew et al., 2010). Its receptor is mainly expressed by T helper 2 cells and mast cells. Frequently released from damaged cells, IL-33 is considered a danger signal (“alarmin”). IL-33 released from mast cells may exert autocrine/paracrine actions on these same cells by augmenting the stimulatory effects of IgE and substance P and by triggering their release of cytokines (Theoharides et al., 2015b). IL-33 is up-regulated in both peripheral leukocytes and CNS of MS patients (Christophi et al., 2012), and IL-33 blockade suppresses development of EAE in C57BL/6 mice during the induction phase (Li et al., 2012). Although IL-33 is expressed by neurons, astrocytes, oligodendrocytes and microglia in human brain, its receptor ST2 is mainly neuronal in location. Acute and chronic MS brain lesion tissues show augmented expression levels of IL-33 and ST2 compared to normal brain (Allan et al., 2016). Further, rat myelinating spinal cord co-cultures treated with IL-33 exhibited inhibition of myelination. MS patients frequently suffer from central neuropathic pain (Osterberg et al., 2005; Solaro et al., 2013). An intriguing possibility is that such pain might involve spinal cord oligodendrocyte-derived IL-33 inducing expression of TNF-α and IL-1β in spinal cord (Zarpelon et al., 2016).

### Amyotrophic lateral sclerosis

Amyotrophic lateral sclerosis (ALS) is neurodegenerative disease that primarily affects upper and lower motor neurons, resulting in progressive muscular paralysis and typically leading to death within 2–5 years of diagnosis. ALS shows clinical, pathological, and genetic overlap with frontotemporal dementia (FTD; Lall and Baloh, 2017). While ALS appears as a composite syndrome with a number of aberrant cellular pathways (Geloso et al., 2017), neuroinflammation is recognized as a key aspect of ALS pathology (Philips and Robberecht, 2011; Liu and Wang, 2017). Activated microglia are a universal feature of ALS/FTD pathology, together with activation of astrocytes at specific disease stages in mouse models of ALS (Hall et al., 1998; Brites and Vaz, 2014; Lee et al., 2016; Lall and Baloh, 2017) and in humans (Turner et al., 2004). Strong evidence points to impairment of the neurovascular unit, including the blood-brain and blood-spinal cord barriers in patients and animal models of ALS (Rodrigues et al., 2012). Mast cells contain preformed TNF-α (unlike glia) and vasoactive mediators, and so may participate in regulating the function of both blood-brain (Ribatti, 2015) and blood-spinal cord barriers. In so doing, mast cells would facilitate entry of immune cells, including themselves (Sayed et al., 2010) across these barriers when compromised, as happens in ischemic stroke and ALS, respectively. Indeed, ALS spinal cord is reported to contain IL-17-expressing (Fiala et al., 2010) and degranulating mast cells. Regulatory T cells enhance mast cell production of IL-6 via surface-bound transforming growth factor-β (Gao and Ji, 2010), which then promotes Th17 activity (Dudeck et al., 2011). Moreover, serum and CSF of ALS patients display elevated amounts IL-12 and IL-15 (Rentzos et al., 2010), the latter cytokine acting as a mast cell chemoattractant (Jackson et al., 2005), while mast cells are a major source of the former (Nakano et al., 2007). IL-12 up-regulates mast cell surface expression of TLR2/TLR4 (Yang et al., 2010), members of a major class of pattern recognition transmembrane receptors activated by PAMPs. PAMPs are molecules associated with groups of pathogens recognized by cells of the innate immune system (Chakraborty et al., 2010). Mast cell (and microglia) TLR2 and TLR4 respond to molecules called damage associated molecular patterns, for example, the high mobility group box 1 protein that is elevated in spinal cord of ALS patients (Casula et al., 2011). Moreover, IL-6 and CCL5 elaborated by microglia could modulate TLR2 and TLR4 expression by mast cells (Pietrzak et al., 2011) to up-regulate chemokines and induce a pro-inflammatory profile in microglia (Skuljec et al., 2011). IL-12 is able as well to up-regulate expression of PAR2 (Zhang et al., 2007), an emerging target for neuroinflammation (Bushell, 2007). Collectively these data point to mast cell-microglia crosstalk, as well as potential autocrine/paracrine actions of cytokines like IL-12 on mast cells.

Mast cell involvement in the neuromuscular junction (NMJ) denervation of ALS was recently investigated in a rat hereditary ALS model (SOD1G93A), where the authors observed a marked infiltration and degranulation of mast cells that started after paralysis onset and correlated with progressive NMJ denervation (Trias et al., 2017). Further, mast cells accumulated around degenerating motor axons and NMJs, and were also associated with macrophages. Mast cell accumulation and degranulation in paralytic muscle was prevented by systemic treatment with masitinib; motor deficits were reduced as well.

### Cerebral ischemia

Eight out of 10 strokes are due to cerebral ischemia, and the remaining ones from cerebral hemorrhage. Stroke is the most common cause of disability, the second commonest cause of dementia and the fourth commonest cause of death in the developed world (Sveinsson et al., 2014) and the leading cause of serious, long-term disability in the United States. Stroke pathology is characterized by an inflammatory response involving microglia activation, cytokine/chemokine release, and macrophage/neutrophil infiltration (Wang et al., 2007; Jordán et al., 2008). Interestingly, in the early phase of the ischemic episode inhibiting microglial cell activation may be of benefit (Hanisch and Kettenmann, 2007), perhaps by phagocytosing debris and/or releasing neurotrophic factors. Another important element in ischemic injury is activation/degranulation of mast cells, a phenomenon that plays a role in initiating the early phase of pathology (Jin et al., 2009; Lindsberg et al., 2010)—even before microglia and astrocyte activation. BBB breakdown accompanies ischemia, allowing for immune inflammatory cell infiltration into brain parenchyma. Mast cells occur within the dura and meninges, and on the brain side of the BBB (Silver and Curley, 2013), and promote BBB breakdown, edema, neutrophil infiltration, and hemorrhage in a rodent model of focal cerebral ischemia (McKittrick et al., 2015). Mast cell degranulation has been observed both in the immature brain after stroke (Biran et al., 2008) and in adult rats undergoing transient global ischemia (Hu et al., 2004). A mast cell role in stroke is strengthened by studies showing that pharmacological mast cell stabilization and genetic mast cell deficiency in rats reduces BBB permeability, brain edema, and neutrophil recruitment (Strbian et al., 2006; Jin et al., 2007; Mattila et al., 2011; Kocic et al., 2015), perhaps by regulating, in part, acute microvascular gelatinase activation (Mattila et al., 2011). Moreover, mast cell blocking limits brain edema and hematoma volume and improves outcome after experimental intracerebral hemorrhage (Strbian et al., 2007). Yet another factor implicated in BBB breakdown is the angiogenic factor vascular endothelial growth factor, which is synthesized, stored, and released by mast cells (Grützkau et al., 2012). In the case of MS, data from EAE models suggest that meningeal (mast cell-mediated) inflammation is a precursor to CNS immune cell (e.g., T cells) infiltration as a consequence of a loss of integrity of both the local BBB and CSF-blood barrier (Sayed et al., 2010; Colloca et al., 2017).

### Traumatic brain injury

Traumatic brain injury (TBI) is a non-degenerative, non-congenital insult to the brain from an external mechanical force that causes brain dysfunction. Mild TBI may cause temporary dysfunction of brain cells. More serious injury can result in bruising, torn tissues, bleeding, and other physical damage to the brain followed by secondary pathological processes including excitotoxicity, ischemia, and neuroinflammation that result in long-term complications and/or death (Hagberg et al., 2012; Xiong et al., 2013). Cognitive and behavioral deficits caused by TBI to the immature brain are more severe and persistent than injuries to the adult brain (Rivara et al., 2012). Among the first events of the injury response, in both the adult and the developing brain, is the degranulation of mast cells (Lozada et al., 2005; Stokely and Orr, 2008) that may facilitate a compromised BBB. In addition, mild TBI evoked by closed head injury is associated with persistent (up to 1 month) dura mast cell degranulation (Levy et al., 2016) and a chronic immune response (Ertürk et al., 2016).

Microglia, together with astrocytes and oligodendrocytes, play instrumental roles in shaping the microenvironment after TBI (Koshinaga et al., 2000; Ramlackhansingh et al., 2011; Karve et al., 2016; Kumar et al., 2017; Taib et al., 2017). In a longitudinal study in humans, chronically activated microglia and tissue degeneration was observed even years after injury (Johnson et al., 2013). Experimental studies in rodents are consistent with the above, showing up-regulation of pro-inflammatory markers (Holmin and Mathiesen, 1999), chronic microglial cell activation, lesion expansion, and hippocampal neuron and myelin loss (Loane et al., 2014). Although often overlooked in the context of TBI secondary injury, also the complement system plays a notable role in this multifaceted inflammatory reaction (Hammad et al., 2018). Astrocytes undergo reactive changes in a setting of TBI, becoming hypertrophic with swelling and extension of processes in the first few days, followed by glial scar formation (Villapol et al., 2014) and with reactive gliosis persisting up to several months post-injury. Although scarring is a potentially a protective mechanism against further injury, it can act to inhibit axonal regrowth and regeneration (Silver and Miller, 2004). Another feature of astrocyte reactivity in TBI is that of proliferation, manifested by an up-regulated expression of glial fibrillary acidic protein, close to the lesion site (Bardehle et al., 2013) and which appears to peak in the acute phase after experimental TBI. As discussed earlier astrocytes, like microglia, can elicit both beneficial and detrimental effects. Moreover, as astrocytes render microglia more responsive to pro-inflammatory stimuli (Barbierato et al., 2013, 2017; Facci et al., 2014) and neurotoxic reactive astrocytes are induced by activated microglia (Liddelow et al., 2017), such behaviors could work in concert the affect the local environment after TBI.

Postoperative cognitive dysfunction shares some features with TBI. Neuroinflammation initiated by extra-CNS surgical trauma, followed by release of CNS inflammatory mediators can damage synapses and neurons and may be a critical component of surgery-induced cognitive dysfunction (Riedel et al., 2014). This phenomenon is often seen in the elderly consequent to surgery and hospitalization (Terrando et al., 2011). As with TBI, cerebral mast cells have been suggested to contribute to postoperative cognitive dysfunction and pain after surgical procedure-mediated neuroinflammation (Oliveira et al., 2011; Li et al., 2017) by promoting BBB breakdown (Zhang S. et al., 2016; Zhang X. et al., 2016). In addition, astrocyte-derived CCL2 participates in surgery-induced cognitive dysfunction and neuroinflammation via evoking microglia activation (Xu et al., 2017).

### Neuropathic pain

Acute and chronic pain are cardinal features of inflammation, albeit different clinical entities. The former is provoked by a specific disease or injury, serves a useful biologic purpose, and is self-limited. Chronic pain, on the other hand, may be considered a disease state that outlasts the normal time of healing and is thought to result from alterations in neuronal cell plasticity. Such alterations include sensitization of peripheral nociceptors in dorsal root and trigeminal ganglia (Basbaum et al., 2009) and central nociceptive neurons in the spinal cord, trigeminal nucleus, brain stem, and cortex (Ossipov et al., 2010). Together, peripheral sensitization and central sensitization translate into a heightened perception of pain. Chronic pain represents a substantial and rising unmet medical need (Smith and Torrance, 2012), and affects 7–10% of the general population (Colloca et al., 2017).

Neuropathic pain represents, without doubt, the most debilitating type of chronic pain, and is a consequence of damage, degeneration, or dysfunction of the sensory nervous system (Jay and Barkin, 2014). Epidemiological studies place a population prevalence of pain with neuropathic characteristics at between 6.9 and 10% (van Hecke et al., 2014), yet it remains largely untreatable. Neuropathic pain is either peripheral or central, as a function of lesion location caused by disease (e.g., diabetes mellitus), medical intervention (chemotherapy, surgery), and injury, the last most often caused by stroke, spinal cord injury, or MS (Kerstman et al., 2013). Peripheral neuropathic pain (painful neuropathy) is, in effect, a brain disease where alterations in neural networks affect multiple aspects of brain function, structure, and chemistry (Borsook, 2012). Analgesics continue to focus on reducing pain transduction and transmission in neurons, which likely accounts for their limited success in controlling disease progression (Ji et al., 2014). This “neuron-centric” view fails to consider that initiation and maintenance of neuropathic pain depend to a great extent on Schwann cells, spinal microglia, and astrocytes, together with elements of the peripheral immune system (Ren and Dubner, 2010) such as mast cells—as will be discussed below.

Mast cells (Héron and Dubayle, 2013) and microglia are frontline protagonists as primary interlocutors for pain neurons, in the periphery as well as at the spinal/supraspinal levels. In the latter case, a new study (Kissel et al., 2017) demonstrates that spinal nerve ligation corresponds temporally and in magnitude with degranulation of thalamic mast cells—a rich source of these cells (Florenzano and Bentivoglio, 2000). Protracted alterations in these immune cells promote persistent neuroinflammation that ultimately impacts neuron functionality. Mast cell mediators like IL-6 activate/sensitize nociceptors which not only contribute to neuropathic pain (Xanthos et al., 2011) but also activate trigemino-cervical and lumbosacral pain pathways, causing widespread tactile pain hypersensitivity (Levy et al., 2012). Peripheral nerve-resident mast cells (and not microglia) are the responders at the site of damage, where they promote recruitment of neutrophils and macrophages (Zuo et al., 2003). In addition, mast cell-derived NGF (Leon et al., 1994) can not only sensitize nociceptors (Kelleher et al., 2016), but mast cells themselves may respond to NGF in a paracrine/autocrine manner. Mast cells could also help in recruiting other immune cell types (e.g., T-cells) which, in turn, release pro-nociceptive mediators. Rats with chronic constrictive nerve injury and treated with glucocorticoids exhibit a reduction in pain and TNF-α-positive mast cell numbers (Hayashi et al., 2011). Mast cells appear to be crucial mediators of chronic visceral pain, as well (Done et al., 2012), and have been proposed as a target in the treatment of complex regional pain syndrome (Dirckx et al., 2013).

Glia are important interlocutors of pain processes at the spinal level (Grace et al., 2014; Old et al., 2015; Echeverry et al., 2017). For example, spinal microglia, upon activation by either cell surface molecules or pro-inflammatory signals released from peripheral immune cells such as mast cells elaborate IL-1β to modulate neuronal cell activity. Dorsal horn microglia become activated in pathological conditions (e.g., peripheral nerve injury) accompanied by up-regulation of ionotropic P2X and metabotropic P2Y purinergic receptors (Kobayashi et al., 2008; Skaper et al., 2010; Biber et al., 2011) to participate in neuropathic pain (Burnstock, 2016; Tsuda, 2016); indeed, inhibiting the function or expression of these microglial receptors strongly attenuates neuropathic pain (Tsuda, 2016). Interactions between mast cells and glia, as will be discussed in a later section, may contribute to amplification of peripheral pain signals at the spinal level. Astrocytes also are a key contributor to neuropathic pain (Milligan and Watkins, 2009; Ji et al., 2013). A recent study by Peng et al. (2016) suggests that microglia and monocytes may act synergistically to promote the transition from acute to chronic pain after nerve injury. Collectively, these findings propose that moderating mast cell-glia reactivity may be a viable therapeutic direction for treating neuropathic pain (Gao and Ji, 2010; Skaper and Facci, 2012; Popiolek-Barczyk and Mika, 2016). In this context it is interesting to point out that acute intracerebroventricular administration of N-palmitoylethanolamine (PEA), a congener of the endocannabinoid anandamide with analgesic and anti-inflammatory activities linked to mast cell/microglia modulation (Alhouayek and Muccioli, 2014; Petrosino and Di Marzo, 2017), reduced carrageenan-induced paw oedema/hyperalgesia (D'Agostino et al., 2007), and chronic pain in man (Paladini et al., 2016).

While not often considered, intriguing evidence suggests that oligodendrocytes, the myelin-producing cells of the CNS, may also participate in pain mechanisms. Among their other roles, oligodendrocytes support, in a myelin-independent manner, axonal functions, and long-term integrity (Nave, 2010; Bankston et al., 2013). Oligodendrocyte ablation causes spinal axonal pathology, along with induction/maintenance of a heightened nociceptive sensitivity in the absence of innate or adaptive immune responses (Gritsch et al., 2014). Further, they produce and respond to chemokines/cytokines that modulate CNS immune responses, express antigen-presenting molecules, complement and complement receptor molecules, complement regulatory molecules, neuroimmune regulatory proteins as well as extracellular matrix proteins (Peferoen et al., 2014; Zeis et al., 2016), and interact with microglia (Peferoen et al., 2014). Intrathecal administration of N,N-dimethylsphingosine (DMS) in rats, whose dorsal horn production is triggered by inflammation, induces neuropathic pain-like behavior (Patti et al., 2012). Human oligodendrocytes produce DMS, and their levels of DMS rise when challenged with agents that damage white matter (Chen et al., 2014). These authors suggest that damage to oligodendrocytes can result in increased DMS production to drive inflammatory astrocyte responses in sensory neuron sensitization. In the case of MS, for example, autoimmune inflammation driven by invading peripheral immune cells may lead to injury/degeneration of oligodendrocytes and neurons, and play a part in the neuropathic pain often experienced by MS patients. In addition, spinal cord oligodendrocyte-derived IL-33 reportedly mediates neuropathic pain (Zhang et al., 2014). von Büdingen et al. (2015) recently demonstrated that NGF directly binds to myelin oligodendrocyte glycoprotein (which shares structural features with TrkA), a protein localized to the outermost lamellae of compact CNS myelin. These authors posit that myelin oligodendrocyte glycoprotein may serve a protective mechanism to remove excess NGF and prevent aberrant sprouting and neuropathic pain after peripheral nerve injury. It is interesting to note that inflammatory cytokines in peripheral nerves have been implicated in the Wallerian degeneration of peripheral nerves after injury and in certain types of inflammatory neuropathies. In analogy to oligodendrocytes (Barbierato et al., 2017), Schwann cells are the primary source of SAA1 production after peripheral nerve injury (Jang et al., 2012).

### Depression

Peripheral immune modulators can induce psychiatric symptoms in animal models and humans. Medical conditions associated with chronic inflammatory and immunological abnormalities, including obesity, diabetes, rheumatoid arthritis, and MS are risk factors for depression (Mezuk et al., 2008; Faith et al., 2011; Matcham et al., 2013; Feinstein et al., 2014). Almost one-half of non-depressed hepatitis C and cancer patients treated with interferon develop depressive symptoms associated with increased serum IL-6 levels (one of the more reliable peripheral biomarkers in major depression) (Loftis and Hauser, 2004), while significantly higher circulating concentrations of TNF-α and IL-6 were reported in depressed subjects compared with controls (Dowlati et al., 2010). Intravenous administration in healthy male volunteers of low-dose endotoxin not only induces a significant increase in peripheral blood concentrations of TNF-α, IL-6, and IL-10 but also results, with some delay, in a selective increase of IL-6 in CSF (Engler et al., 2017). These authors also found a strong association between endotoxin-induced increase of IL-6 in CSF and severity of mood impairment. The cellular mechanisms that underlie depression remain unclear, perhaps due at least in part to the fact that research until now has focused on neuronal cell dysfunction. The role of non-neuronal cells (glia and mast cells especially) in depression has lagged behind. Newer studies indicate that impairment of the normal structure and function of microglia (Prinz and Priller, 2014), caused by either intense inflammatory activation or by decline and senescence of these cells (e.g., during aging), can lead to depression and associated impairments in neuroplasticity and neurogenesis (Brites and Fernandes, 2015; Yirmiya et al., 2015).

Tryptophan catabolism, another important facet of inflammation-induced depression (Hendriksen et al., 2017), involves up-regulation of indoleamine 2,3-dioxygenase (IDO; Maes et al., 2011), the rate-limiting enzyme in the kynurenine pathway. Elevated levels of kynurenine have been linked to depressive-like symptoms in man (Gabbay et al., 2012). As kynurenine has been suggested to enhance IgE-mediated mast cell responses (Kawasaki et al., 2014), it is conceivable that the latter could be affected by alterations in tryptophan metabolism (and, hence, kynurenine levels) (Campbell et al., 2014). Mastocytosis, a rare mast cell activation disorder of both children and adults is characterized by mast cell accumulation in peripheral organs (Valent et al., 2001). Patients with mastocytosis often exhibit not only acute and chronic pain (Wirz and Molderings, 2017) but also psychopathological manifestations such as cognitive impairment; depression appears to be their most common complaint and ranges from 40 to 70% (Rogers et al., 1986; Hermine et al., 2008). Conceivably, systemic brain involvement mediated by mast cell mediators might account for the high prevalence of depression. In this context, mastocytosis patients reportedly display lower levels of tryptophan and serotonin but higher levels of kynurenic and quinolinic acids (Georgin-Lavialle et al., 2016), leading these authors to propose a role for mast cells in the tryptophan pathway leading to depression. Additionally, mast cells have been implicated in mechanisms related to the regulation of emotion (Nautiyal et al., 2008). Masitinib was shown efficacious in treating cutaneous mastocytosis in dogs (Cadot et al., 2011) and in improving recovery from depression associated with mastocytosis (Paul et al., 2010; Moura et al., 2011), suggesting a link between depression in mastocytosis and mast cell activation.

### Autism spectrum disorder

Autism spectrum disorder (ASD) is a life-long condition characterized by marked neurological deficits, especially as relates to cognitive function. Although its pathogenesis remains unknown the major hypothesis at present posits that autism is a multifactorial disorder, possibly being associated to some degree with aspects of autoimmune dysfunction (Theoharides et al., 2013). Mast cells, part of the innate immune system, are reportedly activated in autism (Theoharides et al., 2008a, 2012, 2016), and ASD incidence is claimed to be 10-fold higher in children with mastocytosis (Theoharides, 2009). One such mast cell activator is the neuropeptide neurotensin (Carraway et al., 1982), whose circulating levels are elevated in ASD patients (Tsilioni et al., 2014). A growing body of evidence supports the view that a chronic subclinical inflammation involving both the gut and CNS may contribute to autism symptomatology (Vargas et al., 2005; Thacker et al., 2007; Morgan et al., 2010, 2012; Kern et al., 2016), with active neuroinflammatory processes being found throughout the brain in both cerebral cortex and cerebellum of patients with autism. Intriguingly, a new pair of studies in Nature, by Kim et al. (2017) and Shin Yim et al. (2017) describe how infection during pregnancy increases the risk of neurodevelopmental disorders, such as autism, in offspring. These mouse studies now reveal a link between gut bacteria and atypical brain-circuit connections. Brain abnormalities in persons diagnosed with ASD reportedly show significant ongoing neuroinflammation as a central element of the pathology (Herbert, 2005). Areas of abnormally developed cortex have been identified in individuals with autism (Stoner et al., 2014), and suggest dysregulation of layer formation and layer-specific neuronal differentiation at prenatal developmental stages. Tetreault et al. (2012) reported higher densities of microglia throughout cerebral cortex in brains of people with autism. When sustained, microglial activation can contribute to disease progression and injury of healthy brain tissue through release of pro-inflammatory mediators (Smith et al., 2012) and by engulfing synapses (Rodriguez and Kern, 2011) and other neuronal tissue, thereby leading to cell loss and reduced connectivity, both of which are found in ASD brain (Rodriguez and Kern, 2011).

### Fibromyalgia syndrome

Fibromyalgia syndrome is a prevalent rheumatic disease, that strikes between 2 and 4% of the general population (Queiroz, 2013), predominantly females. This syndrome is characterized by widespread chronic pain, tenderness in muscles and deep tissues, and fatigue/sleep disturbances. The pain of fibromyalgia is a disabling condition and can become quite marked when provoked by digital pressure at tender points. Pain in fibromyalgia is believed to be associated with a generalized alteration (sensitization) in the central somatosensory system (Kim et al., 2015), a condition most likely sustained by neuroinflammatory processes triggered by microglia (Alfonso Romero-Sandoval and Sweitzer, 2015) and mast cell (Kissel et al., 2017) activation. Interestingly, a recent study identified neuropathy of small nerve fibers in patients with fibromyalgia (Üçeyler et al., 2013). Cross-talk between the nervous and immune systems no doubt plays an important role in the initiation and progression of chronic pain in fibromyalgia syndrome and other central sensitivity syndromes (Staud, 2015).

## Neuroinflammation: the risk of growing old

Frailty is a common geriatric syndrome characterized by age-associated declines in physiologic and cognitive reserves across multi-organ systems, resulting in an increased vulnerability for adverse health outcomes (Heuberger, 2011). Chronic (non-resolving) inflammation is likely a key pathophysiologic process that contributes to the frailty syndrome directly and indirectly through other intermediate physiologic systems, and complex multi-factorial etiologies such as obesity and diabetes. Aging is associated with elevated levels of circulating cytokines and pro-inflammatory markers, and age-related changes in the immune system (often referred to as “immunosenescence” or “inflammoaging”) (Michaud et al., 2013; Mate et al., 2014). Hippocampal processing is more easily disrupted in old animals than in younger ones when the peripheral innate immune system is stimulated, suggesting that aging can facilitate neurobehavioral complications associated with peripheral infections (Chen et al., 2008; McManus and Heneka, 2017).

Innate immune cell types, especially mast cells and microglia, are likely to contribute importantly to non-resolving inflammation in the context of aging (Labzin et al., 2018). Although we may think of aging as a general slowing down of the body's cellular activities, the latter cell populations actually appear to become more reactive. For example, as an animal ages, mast cells express alterations in degranulation behavior (e.g., greater sensitivity to prostaglandin E2; Nguyen et al., 2005). Individual microglial cell reactivity/sensitivity appear to persist throughout the entire lifespan, which may explain how stimulation of microglia early in life can induce long-term changes in brain function (Füger et al., 2017). Senescence of resident microglia (and astrocytes) might thus contribute to the age-related increase in risk for neurodegenerative diseases (Hoeijmakers et al., 2016; Labzin et al., 2018; Santoro et al., 2018). In fact, dystrophic (senescent) rather than activated microglia has been noted also in mouse brain with aging (Streit et al., 2009; da Silva et al., 2014; Punzo et al., 2016). Experimental models of aging demonstrate aberrant microglial cell behaviors, in particular in terms of an increased inflammatory state of microglia, in which cells are “primed” to be activated and resistant to regulation (Eggen et al., 2013; Norden and Godbout, 2013; Rawji et al., 2016). Primed microglia are more sensitive to a secondary inflammatory stimulus, thus leading to an exaggerated inflammatory response (Perry and Holmes, 2014). This was demonstrated by culturing microglia-free cerebellar astrocytes together with the addition of increasing numbers of microglia (Facci et al., 2014). The co-cultures displayed a heightened priming response, whereby a classical inflammatory stimulus (lipopolysaccharide, LPS) sensitized (“primes”) the cells to ATP-induced release of IL-1β. Equivalent numbers of microglia alone, however, were essentially devoid of a priming response and IL-1β release. The generality of this phenomenon was shown by the ability of cortical and spinal cord glia to respond to LPS priming in an analogous manner (Facci et al., 2014). These findings serve also to illustrate the concept of glial cell interactions.

Substances other than LPS are capable of effecting a priming behavior. For example, the alarmin high mobility group box 1, released under chronic pathological conditions to initiate inflammatory cascades, mediates neuroinflammatory priming in the aged brain (Fonken et al., 2016). It has also been suggested that microglial priming can be explained by the mechanisms that underlie trained immunity (the latter involving the enhancement of inflammatory responses by epigenetic mechanisms mobilized after first exposure to an inflammatory stimulus) (Haley et al., 2018). When primed, microglia may over-react to a second challenge, resulting in an enhanced pain intensity and duration (Hains et al., 2010). Challenge to the aged brain's immune system leads to amplification/prolongation of microglia activation that may, over a long period of time, manifest itself in deleterious behavioral and cognitive consequences. Xie et al. (2013) found that the age-related decline of myelin proteins is correlated with activation of astrocytes and microglia in rat. In turn, this could disturb microglia clearance function during aging and lead to lysosomal storage, possibly contributing to microglial senescence and immune dysfunction (Safaiyan et al., 2016).

A mast cell—microglia dialogue may likely contribute to exacerbate the effects of aging on their pro-inflammatory behaviors. While not always appreciated as such, both obesity (Theoharides et al., 2015b) and diabetes (Donath, 2014) are states of chronic low-grade inflammation. One might thus predict a general rise in the occurrence of the latter conditions with age, in effect placing the elderly in “harms way” for a condition of low-grade, non-resolving inflammation. Indeed, an endotoxin-induced, persistent state of low-grade inflammation is associated with innate immune “programming” or “memory” (Morris et al., 2015). Given the close link between BBB integrity and cognitive dysfunction in aging (Chan-Ling et al., 2007; Rapp et al., 2008), acute and chronic inflammatory pain states, including neuropathic pain (which is associated with low-grade chronic inflammation), may well alter barrier permeability (Rosenberg, 2012).

## Leveraging endogenous molecules for therapeutic benefit in neuroinflammation

**Figure 4:**
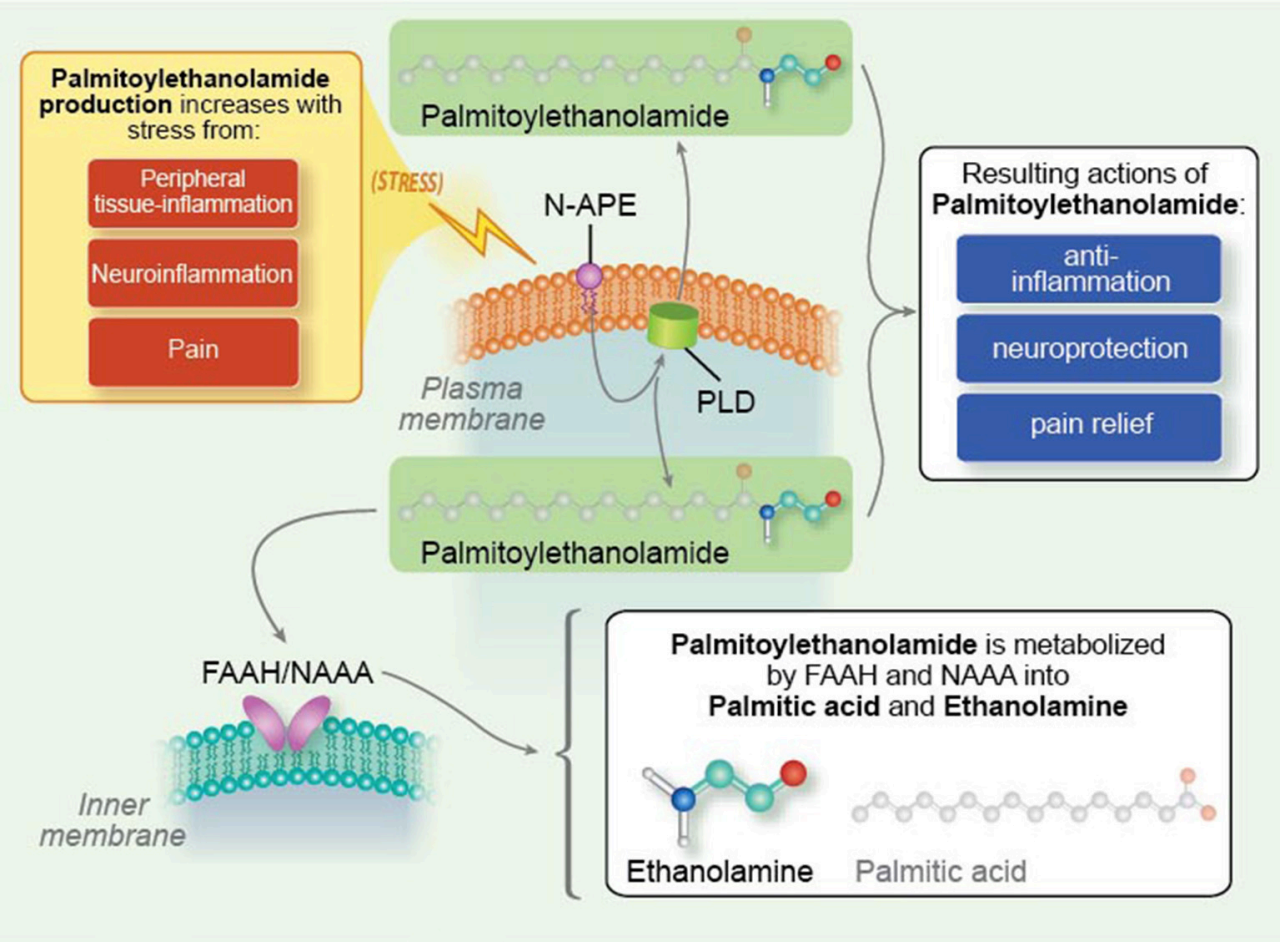
Palmitoylethanolamide synthesis and metabolism. N-palmitoyl-phosphatidyl-ethanolamine (N-APE) is converted into palmitoylethanolamide and phosphatidic acid by a plasma membrane-associated N-acylated phosphatidylethanolamine-phospholipase D (PLD). Palmitoylethanolamide (PEA) is broken down to palmitic acid and ethanolamine by fatty acid amide hydrolase (FAAH, which also catabolizes other fatty acid amides) as well as the more selective N-acyl ethanolamine-hydrolyzing acid amidase (NAAA). Tissue levels of palmitoylethanolamide rise under conditions of stress, e.g., peripheral tissue inflammation, neuroinflammation, and pain [Reproduced from Skaper et al. (2014) Mast cells, glia and neuroinflammation: partners in crime? (Figure 2). Copyright © 2013 John Wiley & Sons Ltd. With permission].

Targeting activation of glia (Gosselin et al., 2010; Möller and Boddeke, 2016; Roser et al., 2017) and mast cells (Graziottin et al., 2014; Hendriksen et al., 2017) is gaining increasing traction as a potential therapeutic avenue for the treatment of nervous system disorders. In addition to small molecule anti-inflammatory agents derived by synthetic chemical routes (Roser et al., 2017), tissue damage and/or stimulation of inflammatory responses activate endogenous protective mechanisms that lead to the elaboration of lipid mediators which function as a program of resolution to switch off inflammation (Buckley et al., 2013; Piomelli and Sasso, 2014). Harnessing such lipid mediators might provide a novel approach to effect a program of resolution (Tabas and Glass, 2013). The mechanism of physiological mast cell regulation, first defined by the Nobel laureate Rita Levi-Montalcini as ALIA (Autocoid Local Injury Antagonism), is the mast cell ability to synthesize on demand the natural mediator PEA. PEA is a member of the N-acylethanolamine (NAE) family, in which a fatty acid is linked to an ethanolamine moiety. NAEs include the endocannabinoid N-arachidonoylethanolamine (anandamide) and its congeners N-stearoylethanolamine, N-oleoylethanolamine and PEA (Pacher et al., 2006). The NAEs (including PEA) are generated principally from by a membrane-associated N-acylated phosphatidylethanolamine (NAPE)-phospholipase D to yield the respective NAE and phosphatidic acid (Figure 4; Leung et al., 2006), although other pathways exist (Ueda et al., 2013). NAEs in the mammalian brain are hydrolyzed to the corresponding fatty acid and ethanolamine by fatty acid amide hydrolase (FAAH; Cravatt et al., 1996) and NAE-hydrolyzing acid amidase (NAAA; Tsuboi et al., 2007; Figure 4). In contrast to FAAH, NAA hydrolyzes NAEs having less than 18 carbon atoms, i.e., PEA (Ueda et al., 2013). The most striking catalytic property of NAAA is a pH optimum at 4.5–5, which is consistent with its immunocytochemical localization in lysosomes (Tsuboi et al., 2007), and with the acidic environment of inflamed tissues.

PEA is produced/hydrolyzed by microglia and mast cells (Bisogno et al., 1997; Muccioli and Stella, 2008), down-modulates mast cell activation (Facci et al., 1995), and controls microglial cell behaviors (Franklin et al., 2003; Luongo et al., 2013). An expanding body of preclinical studies attest to the anti-neuroinflammatory and neuroprotective actions of PEA (Alhouayek and Muccioli, 2014; Esposito et al., 2014; Fidaleo et al., 2014; Mattace Raso et al., 2014; Petrosino and Di Marzo, 2017), and will not be detailed here. It is worth mentioning, however, that PEA decreased aggression in early adolescent socially isolated mice (a putative rodent model of post-traumatic stress disorder; Locci et al., 2017), and rescued learning and memory impairments in a triple transgenic mouse model of AD (Scuderi et al., 2018). These studies were carried out with micronized/ultramicronized PEA, formulations which favor its oral bioavailability over non-micronized PEA (Impellizzeri et al., 2014). Importantly, PEA has proven efficacious in man in a number of clinical settings, which are summarized in Table 3, including a lower frequency of death and tracheotomy in PEA-treated ALS patients compared to untreated patients in terms of the proportion of ALS patients who survived without tracheotomy. None of the clinical trials with PEA to date have reported treatment-related adverse events (Skaper et al., 2014; Paladini et al., 2016).

**Table 3:** Clinical studies demonstrating efficacy of palmitoylethanolamide^a^.

**Pathology**	**References**
Non-surgical lumbar radiculopathies	Chirchiglia et al., 2018
Chronic pain (including neuropathic pain) of differing etiologies	Skaper et al., 2014; Paladini et al., 2016
Endometriosis	Iuvone et al., 2016; Indraccolo et al., 2017
Fibromyalgia	Del Giorno et al., 2015
Parkinson disease (adjuvant therapy)	Brotini et al., 2017
Relapsing-remitting multiple sclerosis (add-on therapy for the treatment of interferon-β1a-related adverse effects)	Orefice et al., 2016
Stroke (adjuvant therapy)	Caltagirone et al., 2016
Amyotrophic lateral sclerosis	Clemente, 2012; Palma et al., 2016
Autism	Antonucci et al., 2015; Bertolino et al., 2017

a *micronized/ultramicronized palmitoylethanolamide*.

Inhibiting the enzymatic degradation of PEA by targeting NAAA, in principle, represents another route in treatment of neuroinflammation. A number of selective NAAA inhibitors have been described (Solorzano et al., 2009; Sasso et al., 2013; Ribeiro et al., 2015; Yang et al., 2015) including systemically active compounds which are able to modulate responses induced by inflammatory stimuli *in vivo* and *in vitro*. The validity of such an approach in man remains to be demonstrated. PEA is not constitutive but is produced on demand, and its catabolic enzymes are probably intended to modulate substrate availability. Given PEA pleiotropic effects, a modulatory (rather than fully inhibitory) approach would maximize availability of the NAE, while assuring that the NAE's component molecules (palmitic acid and ethanolamine in the case of PEA) are returned to the biological system. In principle, this approach should avoid interfering with further on-demand NAE synthesis.

## Epilogue

Inflammation was designed by nature to protect the body in response to injury or infection and promote tissue repair and healing. Uncontrolled, this physiological reaction is transformed into a pathological process that can have profound consequences for nervous system health. Aberrant activation of the innate immune system, in particular involving mast cells and microglia, may occur in either a context-specific fashion or because of the body's inability to resolve a state of protracted inflammation. The former cells are capable of reacting with rapid and longer-term delayed responses, while the latter comprise key sensors for disrupted brain homeostasis and accumulate locally as a consequence of neuronal cell injury or entry of foreign material into the brain parenchyma. We have come to appreciate that a complex interplay exists between non-neuronal cells in the nervous system, including microglia, astrocytes, mast cells and oligodendrocytes. Such cellular behaviors can present a challenge when designing strategies to deal with the resolution of inflammation-associated neurological disorders. This scenario takes on added significance with the knowledge that mast cell and microglia reactivity/responsiveness change with aging. Obesity/metabolic disease and diabetes, for example, with their age-dependent rise in prevalence, can be expected to contribute to low-grade non-resolving inflammation and neuroinflammation. Additionally, acute and chronic inflammatory states may alter BBB permeability and ultimately lead to aging-associated cognitive dysfunction.

We still have much to learn concerning the mechanisms that regulate neuroinflammation. This shortcoming is evident, for example, in current treatments for neuropathic pain; these agents are largely neuron-centric and address the symptoms rather than the underlying pathophysiology. An alternative approach for treating nervous system disorders nay be to focus on endogenous regulators of inflammation. Within this context, the fatty acid amide signaling molecule PEA shows promise by contributing to the resolution of neuroinflammation through modulation of mast cell and glia activity—in other words, a modulator of immuno-neural homeostasis. Molecules capable of modulating activation of both glia and mast cells, without provoking immunosuppression, could be of utility in the resolution of inflammation and restoration of tissue homeostasis.

## Author contributions

All authors contributed to the conception, writing, and critical evaluation of the manuscript. All authors read and approved of the final submitted version of the manuscript.

### Conflict of interest statement

The authors declare that the research was conducted in the absence of any commercial or financial relationships that could be construed as a potential conflict of interest.
