# Corrigendum: An Inflammation-Centric View of Neurological Disease: Beyond the Neuron

**DOI:** 10.3389/fncel.2019.00578

**Published:** 2020-02-03

**Authors:** Stephen D. Skaper, Laura Facci, Morena Zusso, Pietro Giusti

**Affiliations:** Department of Pharmaceutical and Pharmacological Sciences, University of Padua, Padua, Italy

**Keywords:** inflammation, mast cells, microglia, astrocytes, oligodendrocytes, neuro-immune, crosstalk, palmitoylethanolamide

In the original article, there was a mistake in the legend for **Figure 1** as published. We neglected to include the citation for the figure from which our figure was adapted and modified. The correct figure legend appears below.

**Figure 1**. Microglia, like Janus, the two-faced Roman god of beginnings and transitions, display two sides—physiological as well as pathological. While microglial cell activation participates in surveillance that functions to maintain homeostasis and promote synaptic maturation, prolonged exposure to pathogen activators or in settings of systemic inflammation, as may occur in conditions such as diabetes or obesity, can culminate in a state of chronic, non-resolving neuroinflammation. Ultimately, these responses will provoke functional and structural changes and neuronal cell death (neurodegeneration). [Adapted and modified from Heneka et al. (2015). Neuroinflammation in Alzheimer's disease (Figure 1)].

The authors apologize for this error and state that this does not change the scientific conclusions of the article in any way. The original article has been updated.
